# Inadequate sleep is a risk factor for pediatric hypertension: the pathophysiologic and socioeconomic correlates

**DOI:** 10.1038/s41390-025-04199-3

**Published:** 2025-06-25

**Authors:** Alyssa Exarchakis, Sekou Bayo, Anne M. Fink, Diana Martinez

**Affiliations:** 1 Department of Biomedical Sciences, Cooper Medical School of Rowan University, Camden, NJ USA; 2 Department of Biobehavioral Nursing Science, University of Illinois Chicago, Chicago, IL USA

## Abstract

**Abstract:**

The increasing prevalence of pediatric hypertension is concerning to our population because cardiovascular diseases are a leading cause of death in adults. Studies have shown that sleep disturbances in children, such as sleep fragmentation, increase blood pressure and lead to adult hypertension. However, the mechanisms by which this occurs are not fully understood. Most studies regarding sleep disturbances and hypertension are within adults, leaving the adolescent population understudied. In this review, we will describe the physiology of sleep, compare blood pressure variations between adults and adolescents, and discuss social issues regarding sleep fragmentation in children. In addition, we will also discuss the limitations of the studies available and what future research needs to be conducted.

**Impact:**

- Pediatric hypertension is a complex condition that has various underlying causes, thus making treatments complicated.
- We describe the physiology of sleep, compare blood pressure variations between adults and adolescents, and discuss social issues regarding sleep fragmentation in children.
- We synthesize and connect studies to enhance knowledge of the issues that lead to the development of hypertension in pediatrics.
- We show the limitations of previous studies and describe future research that can further our understanding in the development of pediatric hypertension.

## Introduction

Examination of blood pressure (BP) during sleep is possible with the use of ambulatory blood pressure monitoring.^1^ BP follows a circadian rhythm and falls during sleep. The fall in pressure, called the “dip”, is defined as the difference between daytime mean systolic pressure and nighttime mean systolic pressure expressed as a percentage of the day value. A dip of 10–20% is considered normal. Dips less than 10%, referred to as blunted or absent, have been considered as predictors for adverse cardiovascular events, though it is not definitive.^2^ It is important to note that BP is defined differently between adults and youth. Adult hypertension is defined mainly by clinical outcomes such as cardiovascular disease (CVD) risk and mortality. Youth BP is defined from the normative distribution of BP data in healthy children. Table 1 summarizes BP categories and hypertension stages in children.^3^

**Table 1:** Normal and abnormal childhood blood pressure.

Blood pressure category	Age (<13 years of age)	Age ≥13 years of age (mmHg)
Normal Blood Pressure	<90th percentile for age, sex, and height	<120/80
Elevated Blood Pressure	90th–<95th percentile for age, sex, and height	≥120/ < 80 to 129 < 80
Stage 1 Hypertension	≥95th percentile–95th percentile **+** 11 mm Hg	130–139/80–89
Stage 2 Hypertension	≥95th percentile **+** 12 mm Hg	≥140/ ≥ 90

Adapted from: Flynn, J., et al. 2017 and Whelton, P., et al. 2017.

Height, sex, and age are important determinants of pediatric BP. Recent studies show that body size [height, weight, and body surface area (BSA), but not BMI] influences cardiac output and vascular resistance, affecting diastolic blood pressure in young adults. Larger individuals tend to have higher cardiac output and lower vascular resistance when lying down, and greater resistance increases upon standing.^4^ These findings suggest that incorporating body size—especially BSA—into blood pressure assessment could improve accuracy and reduce misdiagnosis of hypertension. Although more research is needed in children, current monitoring standards may benefit from refinement using advanced modeling to account for individual variability. Chronic cardiovascular consequences like left ventricular hypertrophy (LVH) can occur in childhood, although not as common as in adults.^3,5^ In a study assessing longitudinal BP trajectories from childhood and the impact of level-independent childhood BP trajectories on adult LVH, Zhang et al., (2018) reported the association of childhood BP linear slopes with concentric LVH was significantly stronger than that with eccentric LVH during the adolescence period of 12 to 19 years. These observations indicate that the impact of BP trajectories on adult LVH and geometric patterns originates in childhood, highlighting importance for early prevention.^6^ In a meta-analysis assessing the prevalence of LVH and its determinants in children with primary hypertension, only body mass index (BMI) z-score was significantly associated with LVH prevalence (estimate 0.23, 95% CI 0.08–0.39, *p* = 0.004) and accounted for 41% of observed heterogeneity, but not age, male percentage, BMI, or waist circumference z-score. The predominant LVH phenotype was eccentric LVH in patients from specialty clinics (prevalence of 22% in seven studies with 779 participants) and one community screening study reported the predominance of concentric LVH (12%).^7^ These findings provide insight into significant risk factors for LVH in hypertensive youth. In comparison to adults, pediatric hypertension is predominately a sequela of renal pathology.^8^ Globally, pediatric chronic kidney disease (CKD) has been estimated to be 15–74.7 children per million; excluding rates in North America due to lack of data.^9^ In a study investigating left ventricular mass and the factors associated with left ventricular hypertrophy (LVH) in children with stages 2–4 chronic kidney disease, 38% of participants had masked hypertension (normal casual blood pressure but elevated ambulatory blood pressure), while 18% had confirmed hypertension (elevated casual and ambulatory blood pressure). Although there was no significant link between LVH and kidney function, LVH was notably more prevalent in children with confirmed (34%) or masked (20%) hypertension compared to those with normal casual and ambulatory blood pressure (8%). Multivariable analysis revealed that masked hypertension (odds ratio 4.1) and confirmed hypertension (odds ratio 4.3) were the strongest independent predictors of LVH. The authors noted that these findings highlight that casual blood pressure measurements alone are inadequate for predicting LVH in children with CKD. The authors also noted that given the high prevalence of masked hypertension and its correlation with LVH, early echocardiography and ambulatory blood pressure monitoring may be useful in assessing cardiovascular risk in this population.^10^

Although there are differences in the definition of high BP between adults and youth, studies report that pediatric dipping should normally be ≥10%.^11^ While a nocturnal blood pressure dip of over 10% is commonly used as a standard, this threshold has several important limitations. In pediatric populations, it fails to reflect the developing circadian rhythm of blood pressure in children and shows poor reproducibility due to daily variability.^12,13^ It also overlooks individual circadian preferences and the impact of irregular sleep patterns.^14–17^ Additionally, studies have shown that ambulatory blood pressure monitoring itself may disrupt sleep due to cuff inflation, particularly within the first 24 h of use, potentially compromising both sleep quality and the accuracy of the measurements.^18^ However, assessing dipping status is helpful in determining whether adult patients are at risk of cardiovascular events. In adult CKD patients, it is common to have a loss of the typical nocturnal decline in BP by 10%–20% and is associated with LVH and adverse cardiovascular events.^19,20^ Yilmaz et al. (2007) showed that night-time mean systolic BPs, diastolic BPs, and Pittsburgh Sleep Quality Index (PSQI) scores were significantly higher in non-dippers (mean nighttime SBP 136 ± 15, mean nighttime DBP 84 ± 12, percent dipping in SBP,1.4 ± 5.9, percent dipping DBP 2.3 ± 7.4, and PSQI 6.76 ± 3.11) compared with dippers (mean nighttime SBP 123 ± 12, mean nighttime DBP 76 ± 9, percent dipping in SBP 14.3 ± 4.6, percent dipping DBP 16.1 ± 6.5, and PSQI 5.16 ± 2.92). Being a poor sleeper in terms of a high PSQI score (total score>5) was associated with a threefold increased risk of being a non-dipper.^21^ In a separate study evaluating factors affecting circadian BP profile and its association with hypertension-mediated organ damage (HMOD) in pediatric patients with primary hypertension (PH), the nocturnal SBP decrease correlated with BMI Z-score and left ventricular mass index (LVMI) while diastolic DBP decrease correlated with augmentation index (AIx75HR). Patients with a disturbed blood pressure profile (nocturnal drop in SBP or DBP) <10% had the highest LVMI, while extreme dippers (nocturnal drop >20%) had the highest augmentation index (AIx75HR). The authors concluded in pediatric patients with PH, non-dipping is associated with increased left ventricular mass, and extreme dipping may be a risk factor for increased arterial stiffness. Within the same study, non-dipping was found in 44.6% of patients and extreme dipping was found in 25.9% of pediatric patients. These findings highlight the importance of the relationship between poor sleep and cardiovascular function.^22^

Two adult population-based cohort studies found that subjects with intermediate (3–4 ideal metrics) and ideal ( ≥5 ideal metrics) global cardiovascular health (CVH) had lower odds of self-reported sleep-disordered breathing (SDB) in both cohorts.^23^ Although based on self-reported SDB, the study was able to show that poor sleep is associated with poor CVH in humans and increases the risk of cardiovascular disease. A majority of studies available regarding blood pressure and sleep are focused on adults. Youth sleep fragmentation (SF) and its impact on hypertension are understudied and need more research. Although not related to SF, a systematic review of prospective cohorts revealed that elevated BP in childhood or adolescence was significantly associated, in adulthood, with high pulse wave velocity; high carotid intima-media thickness; and left ventricular hypertrophy.^24^ Articles reporting combined effects of child (or adolescent) and adult elevated BP on CVD outcomes in adulthood were also included. In addition to cardiovascular compromise, a systematic review on cognitive testing among children and adolescents with primary arterial hypertension (PAH) reported worse results among individuals with PAH. Results of two prospective trials suggested that cognitive functioning may improve after starting antihypertensive treatment.^25,26^ Significant confounders, namely obesity and sleep apnea, were identified throughout the studies. The review indicates that evidence relating AH with poor cognitive functioning among youth is usually based on indirect measures of executive functions (e.g., questionnaires) rather than objective neuropsychological tests and further studies should be conducted.^27^ These findings highlight the importance of detecting youth hypertension in order to prevent serious adverse events.

The purpose of this systematic review was to determine the mechanisms by which pediatric hypertension occurs and the social/economic effects on pediatric blood pressure. Data from both animal models and clinical studies were examined to understand the effects of inadequate sleep in children and adolescents and the future health consequences in adults. Lastly, we explore the effects of poor sleep in general and discussed improving overall health to combat the increasing incidence of hypertension. A full list of abbreviations used in this manuscript is found in Supplementary Table 1 (S1).

## Developmental changes in sleep wake cycle

**Fig. 1:**
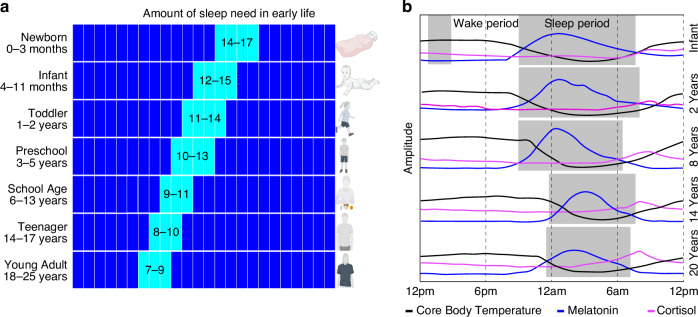
Changes in sleep and physiological processes from newborn to young adulthood. **a**The hours of sleep (aqua) needed decreases from newborn to young adulthood. During the first few months, sleep duration includes frequent napping. The sleep wake cycle is established during the first 6 months of life, during which frequency and duration of naps decreases. **b** 24-h rhythm of the sleep-wake cycle, core body temperature (black), cortisol (magenta) and melatonin (blue) secretion from infancy to 20 years of age. The shortening in sleep duration (gray shading), is accompanied by a delay in the sleep-wake cycle, during adolescence.

The sleep-wake cycle changes throughout development, generally showing a gradual decrease in sleep duration and a shift to a later sleep phase, with more significant changes occurring during adolescence. These alterations are accompanied by changes in neuroendocrine rhythms (e.g., melatonin, cortisol) and circadian rhythms (e.g., body temperature) (Fig. 1).

Throughout infancy and childhood, sleep patterns become more consolidated, leading to fewer and shorter daytime naps. The average sleep duration also steadily decreases over this time, from 12.8 h in infants to 11.9 h for children aged 2–5 years.^28^ This trend (Fig. 1a) continues through childhood, with 6–12-year-olds averaging 9.2 h, adolescents around 16 years old getting 8.1 h, and adults around 24 years old sleeping about 7.5 h.^28–30^

Furthermore, the pineal hormone melatonin plays an important role in regulating sleep wake cycle. It exhibits a 24-h secretion pattern, starting in the evening, peaking during the night, and decreasing in the morning.^31^ This daily rhythm of melatonin strengthens around ages 4–7, and gradually diminishes in intensity throughout one’s lifespan.^32–34^ Cortisol secretion also follows a circadian rhythm, starting with high levels upon waking in the morning, peaking about 30 min later, and gradually decreasing throughout the day to reach its lowest levels around bedtime.^35,36^ During adolescence, cortisol levels rise and the rhythm becomes flatter as age and pubertal development progress.^37,38^ A circadian rhythm of core body temperature (CBT) is established within the first year of life, peaking during the day and dropping to its lowest point at night, a few hours after sleep onset. This rhythm is more pronounced in children than in adults.^37,39–41^ A later timing of the CBT rhythm is linked to eveningness (i.e., later bed and rise times) in adolescents and adults, and the rhythm tends to shift to an earlier phase as people age.^42–44^

## Physiologic consequences of sleep disturbances

Poor sleep has been associated with elevated BP but there are other serious health effects involved as well. In addition to BP changes, a sleep fragmentation (SF) mouse model study demonstrated that long-term SF induces vascular endothelial dysfunction. Elastic fiber disruption and disorganization were also noted in the SF aortas compared to control aortas and fiber disorganization were apparent in SF-exposed mice (*p* < 0.01). Additionally, an increased recruitment of inflammatory cells and altered expression of senescence markers were noted in the SF-exposed mice.^45^

In addition to endothelial dysfunction, changes in the gut microbiome play a role in blood pressure regulation. Using a rat model of SF, Maki et al. (2020) examined the relationship between gut microbiota, blood pressure, and sleep. The SF rats with gut microbiome changes were also found to have significantly higher mean arterial pressure (MAP), systolic and diastolic blood pressure, and heart rate compared to control rats.^46^ These findings suggest that in addition to vascular changes and inflammation, changes in the gut microbiome associated with SF and other sleep disturbances also play a role in the development of hypertension. This animal study highlights potential biochemical mechanisms that lead to cardiovascular compromise and should be studied further.

Sleep disturbances have also been associated with metabolic syndrome (MetS). Metabolic syndrome refers to abnormalities in glucose and lipid metabolism, potentially caused by insulin resistance and/or central obesity. MetS is estimated to affect more than 20% of United States adults, who frequently progress to diabetes and are at increased risk for premature cardiovascular disease.^47^ A study of children with SDB (apnea-hypopnea index ⩾5) showed 6.5 greater odds of MetS compared with children without SDB. Analyses of the individual metabolic parameters showed that SDB was associated with elevated systolic and diastolic blood pressure, low-density lipoprotein cholesterol, and fasting insulin levels after adjustment for body mass index.^48^ Identifying a relationship between sleep and cardiovascular health is of particular interest because the ability to correct sleep disturbances can help prevent serious long-term health conditions, especially in adolescents.

## Adolescents and sleep

Although the data on adolescents and sleep is limited, there are many contributing factors that can lead to disordered sleep in adolescents. Recent studies of adolescents have shown that the delayed internal clock phase is associated with secondary-sex development. This finding suggests a biological component to adolescent changes in sleep patterns. Other mammalian species also show similar changes in the timing of sleep and activity around the development of sexual maturation as evidenced by the review of Hagenauer et al.^49^ There are two mechanisms underlying the changes in the sleep pattern of adolescents: homeostatic drive and circadian timing system. Normally, the circadian timing system promotes sleep in the evening and wakefulness in the morning. The homeostatic drive for sleep or sleep pressure increases the longer the person stays awake and decreases during sleep. However, in adolescence, there is a resistance to sleep pressure which consequently delays the onset of sleep. Additionally, their circadian phase becomes delayed, which causes them to stay awake later in the evening.^50^

Older adolescents have later bedtimes than younger adolescents. This delayed timing of sleep has been attributed to external factors such as evening work,^51^ however, evidence suggests that external factors alone do not completely account for the adolescent delayed sleep onset. The transition into a more evening chronotype—due to a delayed circadian phase—in the second decade of life has been observed across different cultures. This transition is maintained even after weeks of regulated schedules of sleep and under controlled laboratory conditions in which there is limited influence by external factors.^52^ A delayed sleep phase occurs when the sleep pattern becomes delayed two hours or more from conventional or normal sleep patterns; this usually causes a delay in sleep and a later rise time.

Additionally, Taylor et al., used Tanner staging, an objective classification system of pubertal development of secondary sex characteristics to compare different stages in adolescents.^53^ In a study aimed at assessing sleep latency, a measure of sleep tendency (speed of falling asleep), sleep latency after waking did not differ at 20:30 h, but was shorter for the Tanner 1 group at 22:30 h (Tanner 1 = 9.2 ± 6.3 min; Tanner 5 = 15.7 ± 5.8 min), 00:30 h (Tanner 1 = 3.6 ± 1.7 min; Tanner 5 = 9.0 ± 6.4 min), and 02:30 h (Tanner 1 = 2.0 ± 1.7 min; Tanner 5 = 4.3 ± 3.2 min) indicating that more mature adolescents are slower to fall asleep in the evening compared to younger adolescents.^54^ Jenni et al., showed that the homeostatic drive for sleep or the pressure to sleep caused by sleep deprivation was slower in post-pubertal than prepubertal children, reporting a time of 15.4 ± 2.5 h for Tanner 5 adolescents (post-pubertal) and 8.9 ± 1.2 h for those in Tanner stage 1 or 2 (prepubertal).^55^

Both studies suggest that post-pubertal adolescents have a greater tolerance for prolonged waking episodes. Recent evidence suggests that light sensitivity of the circadian system is altered during puberty such that there is an increased sensitivity to the phase-delaying effects of light.^56^ One study found that post pubertal adolescents were significantly less sensitive to dim light exposure in the morning (3:00–4:00) than pre-pubertal adolescents.^57^ These findings suggest that the endogenous circadian system undergoes certain changes at the onset of puberty. However, more research needs to be conducted regarding the association between the observed delay in the circadian phase during puberty.

There are several factors that contribute to insufficient sleep and poor sleep quality in both adolescents and adults including the onset of puberty. US and international studies^58–60^ have reported that adolescents get less sleep as they get older. According to the national sleep foundation, 75% of 12^th^ graders self-reported a sleep duration of less than 8 h, compared to just 16% of 6^th^ graders. This pattern was also observed in other countries as reported by a study in 2005 that more than 1400 South Korean adolescents experienced a reduced sleep duration in high school, with an average sleep duration of 4.9 h.^61^ The decreased sleep duration with increased age among adolescents has been attributed to biologically driven processes due to the onset of puberty. Roenneberg et al.,^62^ reported a 2-h delay for girls and a 3 h delay for boys in the midpoint of sleep—clock time between sleep onset and waking up—across the second decade. Furthermore, the sleep-wake homeostasis, responsible for balancing our need for sleep (sleep pressure or sleep drive) with our need for wakefulness is altered to favor late-night sleep timing as adolescents age.^63^ Recent data seem to indicate that more mature adolescents accumulate sleep pressure at a much slower rate compared to younger adolescents.^55^

Additionally, epidemiologic studies have indicated that individuals from families with low income or of racial or ethnic minorities are at a greater risk of poor quality and insufficient sleep, which contributes to the disparities observed in sleep health.^64,65^ Those with lower socioeconomic status had a less consistent schedule.^66^ Furthermore, several household variables have been identified as factors influencing sleep schedules and quality including overcrowding, noise levels, and safety concerns. Families facing overcrowded living conditions or residing in noisy and unsafe environments may have greater difficulties in maintaining consistent sleep schedules and achieving adequate sleep.^67^ Children from disadvantaged backgrounds often face barriers to accessing quality healthcare, including regular pediatric check-ups and management of chronic conditions. In a study aimed at identifying a connection between area-level socioeconomic indicators and the likelihood of completing or not completing preventative services in pediatric primary care centers, they discovered that patients residing in communities or census tracts with higher poverty rates and limited access to vehicles were approximately 30% less inclined to complete essential preventive services when compared to those living in areas with the lowest poverty rates and highest vehicle access, respectively. This limited access to healthcare services may result in undiagnosed and uncontrolled high blood pressure in children.^68^

It has been reported in numerous studies that simultaneous use of multiple electronic devices is associated with decreased sleep at night. A children’s study in 1976 found that adolescents who watched three or more hours of television had difficulty falling asleep and experienced disrupted sleep throughout the night.^69^ A study of subjects from suburban Philadelphia showed that out of 100 adolescents ranging in age from 12 to 18 years, one-third of the participants had a computer, two-thirds had a television, and 90% had a cell phone in their bedroom. These teenagers were also reported to have simultaneously engaged in an average of four electronic activities after 9 pm.^70^ A possible mechanism postulated for the effects of electronic use on sleep is that it suppresses the release of melatonin due to the light generated by the electronic devices, which consequently disrupts the body’s internal clock.^71^ Several studies have demonstrated that low-intensity light can decrease melatonin secretion at night^72^ and alter circadian rhythms.^73,74^

In addition to its established impact on circadian rhythms and melatonin suppression, evening light exposure has been shown to directly influence cardiovascular physiology, including blood pressure regulation. Importantly, research has demonstrated that exposure to light, particularly in the evening, can acutely elevate blood pressure through non-circadian pathways.^75^ For instance, research indicates that exposure to moderate-intensity light (700 lux, fluorescent) can reduce vagal activity in the autonomic nervous system.^76^ Additionally, exposure to high-intensity light (over 5000 lux, LED) significantly elevates heart rate by enhancing sympathetic nervous system activity.^77^

Moreover, epidemiological evidence supports a broader association between nighttime light exposure and the prevalence of hypertension across different age groups. A study by Obayashi et al. (2014) demonstrated that even low levels of nighttime light exposure ( ≥5 lux) in home settings are associated with increased nighttime systolic and diastolic blood pressure in elderly individuals, highlighting light at night (LAN) as a significant environmental factor affecting cardiovascular health. This association remained significant after adjusting for potential confounding variables, including overnight urinary melatonin excretion and sleep quality, suggesting that the impact of LAN on blood pressure operates independently of circadian hormone regulation and sleep disruption.^78^ In another study, Xu et al. (2023) investigated the relationship between objectively measured bedroom LAN exposure and blood pressure in a sample of Chinese young adults aged 16–22 years. The study found that higher levels of LAN were significantly associated with elevated SBP and a greater risk of hypertension, even in this relatively healthy, young population. Specifically, each 1 lux increase in bedroom LAN intensity corresponded to a 0.55 mmHg increase in SBP.^79^ These observations suggest that in addition to sleep disruption, light exposure may serve as a direct environmental risk factor for pediatric hypertension.

Moreover, although caffeine use in adolescents has not been extensively studied, it has been reported to have deleterious effects on their sleep patterns. Caffeine consumption among adolescents is associated with shorter sleep duration, increased sleep onset latency, and increased wake time after sleep onset.^80–82^ In 2008, Roehrs and Roth concluded that high and regular caffeine users experienced disrupted sleep, which led to more daytime sleepiness and that in turn led to more caffeine consumption during the day.^83^

## Adolescent sleep disturbances and hypertension

There are several modifiable and non-modifiable confounders in the development of hypertension in adolescents (Table 2,^84–89^). Javaheri et al. conducted a cross-sectional study that assessed whether insufficient sleep is associated with prehypertension in healthy adolescents.^90^ After adjustment for the same variables, those with polysomnography sleep efficiency ≤85% had nearly 3 times the odds of prehypertension as those with better sleep (OR, 2.83; 95% CI, 1.28, 6.24). However, it is important to note that the correlation between actigraphy sleep efficiency and PSG sleep efficiency was low (r = 0.13, *p* = 0.04). Approximately one-third (32.8%) of adolescents with low sleep efficiency as assessed on actigraphy also had low sleep efficiency from the polysomnography. In a study comparing actinography and PSG, sleep onset latency (SOL) and wakefulness after sleep onset (WASO) measurements had some disagreement.^91^ After Bland-Altman concordance between actinography and PSG, the disagreement between measurement modalities increased as the average value of SOL or WASO increased. This indicates that actigraphy may be less accurate by falsely reporting long periods of quiet wakefulness as sleep (shorter SOL) or reporting body movements for disturbed sleep (longer SOL). While actigraphy is an accurate method for measuring sleep, PSG remains the gold standard. Within the same study, the authors also compared sleep diaries to actinography and PSG. Sleep diaries, which are a form of subjective sleep measurement, had statistically significant differences for all variables with actinography and PSG.^91^ Although subjective measures such as surveys and diaries are inexpensive and accessible, they are less accurate and more biased. A variety of methods are available to assess sleep in children and adolescents, each with specific strengths, limitations, and criteria (see Table 3 for a detailed comparison).

**Table 2:** Modifiable and non-modifiable risk factors in juvenile and adolescent hypertension.^71–76^

Non-modifiable	Modifiable
Socioeconomic status	Diet (excess salt intake)
Genetics	Sleep disorders (sleep disordered breathing and obstructive sleep apnea)
Family history (parental hypertension, premature birth, low birth weight, second-hand smoke exposure)	Overweight/obesity
Gender	Medications (oral contraceptives, antidepressants, bronchodilators, steroids, etc)
Endocrine/ Hormonal issues (diabetes, metabolic syndrome)	Physical activity
Racial/Ethnicity predisposition	
Mental Illness (depression, anxiety)

**Table 3:** Methods to assess sleep in children and adolescents: strengths, limitations, and criteria.

Method	Description	Strengths	Limitations	Criteria
**Polysomnography (PSG)**	Comprehensive sleep study that records brain waves, oxygen levels, heart rate, and breathing during sleep.	Gold standard for diagnosing sleep disorders.	Expensive, requires specialized equipment and overnight stay.	Used to diagnose OSA, sleep efficiency <85% indicates poor sleep quality.
**Actigraphy**	Wearable device that measures movement, often used to estimate sleep patterns over multiple days.	Non-invasive, inexpensive, provides long-term sleep data.	Less accurate than PSG; can misinterpret periods of quiet wakefulness as sleep.	Worn continuously for at least 24 h. Activity is usually recorded for a period of 3 days to 2 weeks
**Sleep Diaries**	Subjective self-report or caregiver report of sleep patterns and behaviors.	Inexpensive and easy to administer.	Highly subjective and prone to recall bias.	Requires consistent and accurate reporting for valid results.
**Ambulatory BP Monitoring**	Monitors blood pressure over 24 h, including during sleep, to assess circadian BP variations.	Objective data on BP changes during sleep, helps assess hypertension risks.	Requires equipment, uncomfortable for continuous wear.	Dipping status ( ≥ 10%) assessed as a predictor for cardiovascular risk.

### Obstructive sleep apnea (OSA) and hypertension

In another study, blood pressure was measured during polysomnography in 41 children with obstructive sleep apnea (OSA) and compared to 26 children with primary snoring (PS).^92^ OSA is characterized by episodes of complete collapse of the airway or partial collapse with an associated decrease in oxygen saturation or arousal from sleep resulting in fragmented, nonrestorative sleep.^93^ The results showed that children with OSA had a significantly higher diastolic BP than those with PS (*p* < 0.001 for sleep and *p* < 0.005 for wakefulness). In adults with OSA, symptoms typically include daytime somnolence whereas children with OSA are more likely to present with behavioral and cognitive disorders, including hyperactivity, attention-deficit disorder, poor school performance, and nocturnal enuresis.^94^ OSA diagnosis is based on polysomnography (PSG) results. According to the 2012 AASM scoring manual, the criteria for events during sleep for infants and children can be used for those who are 18 years younger of age, but individual sleep specialists can choose to score children who are 13 years of age or older using adult criteria. The severity of OSA can be categorized by the apnea/hypopnea index (AHI). In a study assessing the predictors for pediatric hypertension, late childhood/adolescence, obesity, and severe OSA were independent predictors. Furthermore, late childhood/adolescence, and abnormal SpO_2_ (mean SpO_2_ < 95%) independently predicted hypertension in obese children. Severe OSAS independently predicted hypertension in non-obese children.^95^ Although PSG is the gold standard, it is important to note that the PSG and BP measurements are often recorded for one night and in a clinical research building which could contribute to changes in blood pressure and sleep. Additionally, one recording may not represent the true blood pressure or sleep parameters due to variability in individual sleep specialist scoring children based on age.

In a longitudinal study by Chan et al. (2020), childhood moderate-to-severe OSA was associated with higher nocturnal systolic blood pressure (SBP) (difference from normal controls: 6.5 mmHg, 95% CI 2.9–10.1) and reduced nocturnal dipping of SBP ( − 4.1%, 95% CI − 6.3% to 1.8%) at follow-up, adjusted for age, sex, BMI and height at baseline, regardless of the presence of OSA at follow-up.^96^ Childhood moderate-to-severe OSA was also associated with higher risk of hypertension (relative risk (RR) 2.5, 95% CI 1.2–5.3) and non-dipping of nocturnal SBP (RR 1.3, 95% CI 1.0–1.7) at follow-up.

In another longitudinal study by Fernandez-Mendoza et al. (2021), persistent apnea-hypopnea index (AHI) of 2 or more since childhood was longitudinally associated with adolescent elevated BP (eBP) (odds ratio [OR], 2.9; 95%CI 1.1–7.5), while a remitted AHI of 2 or more was not (OR, 0.9; 95%CI 0.3–2.6). EBP is considered >90^th^ percentile but less than the 95^th^ percentile.^97^ Adolescent OSA was associated with eBP in a dose-response manner; however, the association of an AHI of 2 to less than 5 among adolescents was nonsignificant (OR, 1.5; 95% CI, 0.9–2.6) and that of an AHI of 5 or more was approximately 2-fold (OR, 2.3; 95%CI, 1.1–4.9) after adjusting for visceral adipose tissue. An AHI of 5 or more (OR, 3.1; 95%CI, 1.2–8.5), but not between 2 and less than 5 (OR, 1.3; 95%CI, 0.6–3.0), was associated with orthostatic hyperreactivity among adolescents even after adjusting for visceral adipose tissue. Childhood OSA was not associated with adolescent eBP in female participants, while the risk of OSA and BP was greater in male participants.

The association between OSAS, decreased sleep duration and higher risk of hypertension is supported by data from other countries. In a retrospective cross-sectional study by Chuang et al. (2021), conducted in Taiwan, they investigated how obesity and OSAS severity affect BP in children.^95^ There was a trend of an increasing prevalence of hypertension in the children with more severe OSAS (*p* < 0.001), 20.9% and 38.2% in mild and severe OSAS group respectively. Furthermore, in the overall cohort, OSAS severity was found to be an independent predictor of pediatric hypertension. However, upon subgroup analysis, the effects of OSAS severity varied among patients with different weight status. In the non-obese children, OSAS severity was the only predictor of hypertension (OR = 2.18, 95% CI = 1.17–4.05, *p* = 0.014). Whereas, among obese children, OSAS severity did not independently predict hypertension (OR = 1.59, 95% CI = 0.65–3.92, *p* = 0.313).

In a cross-sectional study conducted by Bal et al. (2018), the researchers examine the association between blood pressure and the duration of sleep within primary and secondary schools in Turkey.^98^ Based on the results of the univariate binary logistic regression analyses, the study determined that having a sleep duration of less than 8 h is a notable risk factor for hypertension in both males and females. Furthermore, for both boys and girls, with each additional hour of sleep, the risk of elevated blood pressure decreased (OR:0.89, CI:0.82–0.98 for boys) and (OR:0.88, CI: 0.81–0.97 for girls). In multiple binary logistic regression analyses (adjusted for age and BMI), the analyses revealed that shorter sleep duration remained a risk factor for elevated blood pressure. The various causes of sleep disturbances, including obstructive sleep apnea, sleep fragmentation, and inadequate sleep duration, are key factors in pediatric hypertension (see Table 4 for definitions, impacts, and their link to hypertension).

**Table 4:** Causes of sleep disturbances in children and adolescents: definitions, impact, and link to hypertension.

Cause	Definition	Impact on sleep	Link to hypertension
Obstructive Sleep Apnea (OSA)	Recurrent episodes of upper airway obstruction during sleep, leading to apnea/hypopnea.	Causes fragmented sleep, oxygen desaturation, and daytime fatigue.	Increases risk of pediatric hypertension due to disrupted sleep and intermittent hypoxia.
Duration of Sleep	Total time spent sleeping per night, including naps.	Inadequate sleep duration contributes to poor cognitive development and metabolic disturbances.	Shortened sleep duration has been associated with elevated blood pressure in children and adolescents.
Sleep Fragmentation	Repeated interruptions of sleep, causing poor sleep quality despite adequate duration.	Reduces restorative sleep and increases daytime sleepiness.	Sleep fragmentation has been linked to endothelial dysfunction, increased blood pressure, and cardiovascular risk.

### The influence of socioeconomic and environmental factors on pediatric blood pressure

Socioeconomic factors may be another determinant of pediatric blood pressure and have been found to significantly impact pediatric blood pressure. East et al. (2020) showed that children from lower socioeconomic backgrounds exhibited higher blood pressure levels compared to their peers from higher socioeconomic backgrounds.^99^ The researchers attributed this association to various factors, including limited access to healthcare services, unhealthy dietary habits, and increased exposure to psychosocial stressors.

Bal et al., also looked at various environmental conditions and their relationship to elevated blood pressure and found that living in urban as opposed to rural areas was as a risk factor for an increased risk of prehypertension and hypertension, OR: 1.43, CI: 1.11–1.84 for boys and OR: 1.77 CI:1.37–2.27 for girls (*p* < 0.05). There was no relationship between maternal employment, paternal education, house size, elevator use and mode of transport to school (*p* > 0.05).

## Discussion of overall consequences of poor sleep

Although difficult to control, an issue worth highlighting is the different age ranges used to categorize adolescents. It is important to note that many of the studies conducted on adolescents generally do not utilize a standardized age range (Table 5). The range in ages has a significant impact because intrinsic factors like pubertal status seem to play a role in the development of hypertension. In addition, external factors such as schoolwork, extracurricular activities, home-life, electronic use can play a role in the adolescents’ sleep schedules and future studies should collect and examine this information as it could increase the power of these studies findings. The consequences of sleep insufficiency and disturbance are numerous, including a lack of higher-level cognitive skills, the development of which is critical during adolescence. Chronic sleep loss has been associated with increased alcohol and drug use.^100^ This observation however may be bidirectional as it has also been reported that alcohol consumption can lead to insufficient and poor-quality sleep.^101^ Sleep deprivation has also been associated with depressive moods. Sleep-deprived college students are reported to experience a higher risk of depressive symptoms,^102^ the same observation has been made in high school students.^103^ Additionally, several studies have linked sleep insufficiency to suicidal ideation. Adolescents who sleep less than 8 h at night are three times more likely to attempt suicide.^104,105^ The American Academy of Sleep Medicine has established fresh consensus guidelines regarding the recommended amount of sleep essential for maintaining overall health, with a focus on preventing hypertension in children and adolescents.^106^ As per these guidelines, teenagers aged 13–18 should aim for 8–10 h of sleep within a 24-h period consistently to support their optimal health.

**Table 5:** Summary of studied included in the present literature review and narrative description.

Year	Authors	Number of Participants	Age (years)	Methods	Results
2008	Javaheri, S.,Storfer-Isser A., Rosen, C. L., & Redline, S.	238	13–16	Cross-sectional analysis of 238 adolescents, all without sleep apnea or severe comorbidities. Participants underwent multiple-day wrist actigraphy at home to provide objective estimates of sleep patterns. In a clinical research facility, overnight polysomnography, anthropometry, and 9 blood pressure measurements over 2 days were made. Exposures were actigraphy-defined low weekday sleep efficiency, an objective measure of sleep quality (low sleep efficiency ≤85%), and short sleep duration ( ≤6.5 h).	In unadjusted analyses, the odds of prehypertension increased 4.5-fold (95% CI, 2.1 to 9.7) in adolescents with low sleep efficiency and 2.8-fold (95% CI, 1.1–7.3) in those with short sleep. In analyses adjusted for sex, body mass index percentile, and socioeconomic status, the odds of prehypertension increased 3.5-fold (95% CI, 1.5. 8.0) for low sleep efficiency and 2.5-fold (95% CI, 0.9–6.9) for short sleep. Adjusted analyses showed that adolescents with low sleep efficiency had on average a 4.0 ± 1.2-mm Hg higher systolic blood pressure than other children (*P* < 0.01).
1998	C.L. Marcus, M.G. Greene, and J.L. Carroll.	67	OSAS group: 5 ± 3 PS group: 8 ± 4*	Measured blood pressure (BP) during polysomnography in 41 children with OSAS, compared to 26 children with primary snoring (PS). Systolic and diastolic BP were measured every 15 min via an appropriately sized arm cuff, using an automated system. This was tolerated by the children without inducing arousals from sleep.	Children with OSAS had a significantly higher diastolic BP than those with PS (*p* < 0.001 for sleep and *p* < 0.005 for wakefulness). There was no significant difference in systolic BP between the two groups. Multiple linear regression showed that blood pressure could be predicted by apnea index, body mass index, and age. Blood pressure during sleep was lower than during wakefulness (*p* < 0.001 for diastole and *p* < 0.01 for systole), but did not differ significantly between rapid eye movement (REM) and non–REM sleep.
2007	S. Redline, A. Storfer-Isser, C.L. Rosen, N.L. Johnson, H.L. Kirchner, J. Emancipator, and A.M. Kibler.	270	13.6 ± 0.7 years	Standardized measurements of SDB, anthropometry and bioassays, were made in 270 adolescents, aged 13.6 ± 0.7 years. MetS was identified if threshold levels were exceeded in three of five areas: waist circumference, blood pressure, triglyceride level, high-density lipoprotein cholesterol level, and glucose levels.	Although 70% of children with SDB (apnea–hypopnea index ⩾ 5) were overweight and 59% had MetS, 16% of children without SDB had MetS. Twenty-five percent of those with MetS had SDB. After adjusting for age, race, sex, and preterm status, children with SDB had a 6.49 (95% confidence interval, 2.52, 16.70) increased odds of MetS compared with children without SDB. Indices of SDB stress associated with MetS included respiratory event frequency, degree of oxygen desaturation, and sleep efficiency. Analyses of individual metabolic parameters showed that, after adjustment for body mass index, SDB was associated with systolic and diastolic blood pressure, low-density lipoprotein cholesterol, and fasting insulin levels.
2009	Javaheri, S.,Storfer-Isser A., Rosen, C. L., & Redline, S.	238	12–18	Subjects were recruited from a pediatric office in a proximal suburb of Philadelphia, Pennsylvania. Inclusion criteria for this study were middle and high school subjects aged 12 to 18 years old. The questionnaire, Adolescent Sleep, Caffeine Intake, and Technology Use, was developed by the investigators to measure adolescents’ intake of caffeinated drinks, use of nighttime media-related technology, and sleep behaviors. Descriptive statistics characterized the subjects, their caffeine and technology use, and sleep variables. Regression models assessed the relationships between caffeine, technology use, and sleep variables, having adjusted for age, race, gender, and BMI.	Sleep was significantly related to the multitasking index. Teenagers getting 8–10 h of sleep on school nights tended to have 1.5- to 2-fold lower multitasking indices compared with those getting less sleep. Thirty-three percent of the teenagers reported falling asleep during school. Caffeine consumption tended to be 76% higher by those who fell asleep. The log-transformed multitasking index was significantly related to falling asleep during school and with difficulties falling asleep on weeknights.
2006	Crowley, S., Acebo,C., Fallone, G., Carskadon, M.	149	9–17	One group, ages 9–17 years (mean age = 12.5, SD = 2.3 years, 74 males, 75 females) contributed 149 DLMO phase and sleep/wake pattern measures while on a school year schedule (“school group”). A separate group, ages 9 to 16 years (mean age = 13.1, SD = 1.3 years, 30 males, 29 females) contributed 59 DLMO phase and sleep/wake pattern measures while on a summer schedule (“summer group”).	Bedtime, midsleep time, and wake-up time were positively correlated with DLMO phase in both groups. Although all correlation coefficients for the summer group were statistically greater compared to the school group, the regression equations predicted DLMO phase within ±1 h of the measured DLMO phase in approximately 80% for both groups
2005	Taylor,D., Oskar, J., Acebo, C., Carskadon, M.	20	11.1–13.9	A sample of 20 children was selected from those who participated in studies of circadian timing that included an episode of extended waking. Sample selection was based upon pubertal stage at the time of study, such that participants were either prepubertal or fully mature. The sample included nine prepubertal (pubertal stage Tanner 1, mean age 11.1 years, SD ± 1.3years, five males) and 11 mature adolescents (Tanner 5, 13.9 ± 1.2 years, three males).	The mean time of DLMO in the Tanner 1 group was significantly earlier (mean clock time = 20:33 h; SD = 49 min) than in the Tanner 5 group (21:29 h; 42 min) as determined by a two-tailed t-test (t = −2.78; df=18; *P* = 0.01).
2005	Jenni, O., Achermann, P., Carskadon, M.	13	11.9–14.2	Subjects were studied during baseline and recovery sleep after 36 h of wakefulness. Seven prepubertal or early pubertal children (pubertal stage Tanner 1 or 2 = Tanner 1/2; mean age 11.9 years, SD + /− 0.8, 2 boys) and 6 mature adolescents (Tanner 5; 14.2 years, ± 1.4, 2 boys). All-night polysomnography was performed. EEG power spectra (C3/A2) were calculated using a Fast Fourier transform routine.	In both groups, sleep latency was shorter, sleep efficiency was higher, non-rapid eye movement (NREM) sleep stage 4 was increased, and waking after sleep onset was reduced in recovery relative to baseline sleep. Spectral power of the NREM sleep EEG was enhanced after sleep deprivation in the low-frequency range (1.6–3.6 Hz in Tanner 1/2; 0.8–6.0 Hz in Tanner 5) and reduced in the sigma range (11–15 Hz). Sleep deprivation resulted in a stronger increase of slow-wave activity (EEG power 0.6–4.6 Hz, marker for sleep homeostatic pressure) in Tanner 5 (39% above baseline) than in Tanner 1/2 adolescents (18% above baseline). Sleep homeostasis was modeled according to the two-process model of sleep regulation. The build-up of homeostatic sleep pressure during wakefulness was slower in Tanner 5 adolescents (time constant of exponential saturating function 15.4 + /− 2.5 h) compared with Tanner 1/2 children (8.9 + /− 1.2 h). In contrast, the decline of the homeostatic process was similar in both groups.
2010	Huang, Y., Wang, C., Guilleminault, C.	1939	12–13, 14–16,17–18	This is a cross-sectional, community based study with self-reported sleep questionnaires. Completed questionnaires from 1939 adolescent subjects from schools in Lin-Kou district (Taipei, Taiwan) (96.7% responded); 1906 valid questionnaires (62.3% girls) were analyzed. The randomly selected classes included elementary grade 6 (age range: 12–13 years), junior high school (age range: 14–16 years) and senior high school students (age range: 17–18 years).	The mean sleep duration on weekdays was 7.35 ± 1.23 h and on weekends 9.38 ± 1.62 h. Weeknight sleep decreased significantly with increasing school grade (6.87 ± 1.14 h for high school seniors). There was a trend towards increased daytime sleepiness for students in higher school grade levels. Daytime sleepiness directly correlated with shorter total sleep time (TST) on weekdays, longer TST on weekends, snoring, insomnia and nightmares. Coffee intake, smoking, periodic leg movement/restless legs syndrome, body mass index (BMI), mouth breathing and breathing problems were indirect factors that induced daytime sleepiness. Pearson correlation showed no significant correlation between the TST during the weekday and BMI (–0.047, *p* = 0.079) or body weight (BW) (–0.048, *p* = 0.072). But it showed significant negative correlation (–0.103, *p* = 0.0001) for increasing total sleep time on the weekend and decreasing BMI.
2020	Gariepy,S., Danna, S., Gobina, I., Rasmussen, M., Gasper de Matos, M., Tynjälä,J., Janssen, I., Kalman, M., Villeruša,A., Husarova,D., Brooks, F., Elgar, F., Klavina-Makrecka, S., Šmigelskas,K., Gaspar,T., Schnohr,C.	165,793	13.5	We obtained sleep data on 165,793 adolescents (mean age 13.5 years; 50.5% girls) in 24 European and North American countries from the recent cross-sectional Health Behavior in School-aged Children surveys (2013–2014 and 2017–2018). For each country, we calculated the age-standardized mean in sleep duration, timing, and consistency and the proportions meeting sleep recommendations on school and nonschool days from self-reported bedtimes and wake times. We conducted stratified analyses by gender, age, and family affluence group.	Adolescent sleep patterns varied cross-nationally. The average sleep duration ranged between 7:47 and 9:07 h on school days and between 9:31 and 10:22 h on nonschool days, and the proportion of adolescents meeting sleep recommendations ranged between 32% and 86% on school days and between 79% and 92% on nonschool days. Sleep patterns by gender and affluence groups were largely similar, but older adolescents slept less and went to bed later on school days than younger adolescents in all countries.
2005	Yang, C., Kim, J., Patel, S., Lee, J.	1457	13.7	The School Sleep Habits Survey was administered in homeroom classes to students in grades 5 to 12 (mean age: 13.7 + /- 2.4 years) selected via a 2-way stratification sampling method. The survey included items regarding usual sleep/wake patterns over the previous 2 weeks as well as measures of daytime sleepiness, sleep/wake-problem behavior, depressed mood, and morningness/eveningness.	A total of 1457 students (52.9% male) completed the survey. The higher the grade, the later bedtime was found to be on both school days and weekends. There was a similar relationship between increasing grade and earlier wake time on school days, but higher grades were associated with later wake time on weekends. Total sleep time decreased by approximately 3 h on school nights and 1 h on weekend nights from grades 5 to 12. Adolescents were severely sleep deprived, with mean school-night total sleep times of 6.02, 5.62, and 4.86 h for 10th-, 11th-, and 12th-graders, respectively. In the higher grades, there was a greater discrepancy between school nights and weekends in terms of bedtime and wake time, and the magnitude of weekend oversleep increased. Older students also reported more daytime sleepiness, more sleep/wake-problem behavior, more depressed mood, and more eveningness preference. The chief reasons students cited for their sleep deprivation differed across grades: Academic demands and entertainment (such as Internet and television) were reported by 5th- and 6th-graders, entertainment and then academic demands by 7th-, 8th-, and 9th-graders, and early school start time and academic demands by 10th-, 11th- and 12th-graders.
2010	Patel, N., Grandner, M., Xie, D., Branas, C., Gooneratne, N.	9714		A cross-sectional survey of 9714 randomly selected subjects was used to explore sleep quality obtained by self-report, in relation to socioeconomic factors including poverty, employment status, and education level. The primary outcome was poor sleep quality. Data were collected by the Philadelphia Health Management Corporation	Significant differences were observed in the outcome for race/ethnicity (African-American and Latino versus White: unadjusted OR = 1.59, 95% CI 1.24–2.05 and OR = 1.65, 95% CI 1.37–1.98, respectively) and income (below poverty threshold, unadjusted OR = 2.84, 95%CI 2.41–3.35). In multivariable modeling, health indicators significantly influenced sleep quality most prominently in poor individuals. After adjusting for socioeconomic factors (education, employment) and health indicators, the association of income and poor sleep quality diminished, but still persisted in poor Whites while it was no longer significant in poor African-Americans (adjusted OR = 1.95, 95% CI 1.47−2.58 versus OR = 1.16, 95% CI 0.87–1.54, respectively). Post-college education (adjusted OR = 0.47, 95% CI 0.31–0.71) protected against poor sleep.
2004	Spilsbury, J., Storfer-Isser, A., Drotar, D., Rosen, C., Kirchner, L., Benham, H., Redline, S.	755	8.0–11.0	Cross-sectional analysis of 755 (50% female, 35% ethnic minority) children 8 to 11 years old from a community-based sample of children participating in a cohort study. Sleep and health data were obtained from a child-completed 7-day sleep journal and a caregiver-completed health/sleep questionnaire	Mean (SD) sleep duration for all children was 9.63 (0.72) h. Univariate results showed a statistically significant decrease in mean sleep duration associated with increasing age (*P* < 0.001) and male sex (*P* = 0.03). At all ages, minority boys slept significantly less than nonminority boys and girls and minority girls. The shortest covariate-adjusted mean sleep duration was observed among the oldest minority boys (9.28 [0.07] hours vs 9.43–9.85 h in the other age, sex, and ethnicity subgroups). Forty-three percent of 10- to 11-year-old minority boys reported less than 9 h nightly sleep vs 5% to 26% of the other age, sex, and ethnicity subgroups. After controlling for potential confounding, minority children were more likely than nonminority children to have a bedtime of 11 pm or later (odds ratio, 4.8; 95% confidence interval, 2.9–8.0).
2016	Jones, M. N., Brown, C. M., Widener, M. J., Sucharew, H. J., & Beck, A. F.	5298		This was a retrospective review of the electronic health record for 5298 infants born consecutively between May 1, 2011 and November 30, 2012 and seen in 1 of 3 primary care centers. Each infant was followed for 15 months. Exclusion of patients with street addresses outside the primary service area of Hamilton and Butler Counties, and those with invalid address data, resulted in a final sample size of 4872 infants. Addresses were geocoded in ArcGIS 10.2 (Redlands, CA) using the address locator toolbox and street data from 2005 maintained by ESRI and TeleAtlas, linking every patient record to a precise geographic location representing their residence. Patients were then connected to the census tract in which the address was located; tract-level socioeconomic variables were appended to the patient record.	We found significant associations between area-based socioeconomic measures and completion patterns of recommended preventive services. Patients living in communities, or census tracts, with higher rates of poverty and lower rates of vehicle access were ~30% less likely to complete key preventive services compared with the lowest poverty and highest vehicle access quartiles, respectively. Such area-based data could be applied at the patient-level to improve completion of preventive services via patient-level risk stratification and tailoring of interventions. Such data could be similarly applied at the primary care center or population level to more effectively target preventive service delivery. Such strategies are especially relevant given renewed focus on preventive service delivery.
2004	Johnson, J., Cohen, P., Kasen, S., First, M., Brook, J.	759	14–22	A community-based sample of 759 mothers from upstate New York and their offspring were interviewed during the early adolescence (mean age, 14 years), middle adolescence (mean age, 16 years), and early adulthood of the offspring (mean age, 22 years).	Adolescents who watched 3 or more hours of television per day during adolescence were at a significantly elevated risk for frequent sleep problems by early adulthood. This elevation in risk remained significant after offspring age, sex, previous sleep problems, offspring psychiatric disorders, offspring neglect, parental educational level, parental annual income, and parental psychiatric symptoms were controlled statistically. Adolescents who reduced their television viewing from 1 h or longer to less than 1 h per day experienced a significant reduction in risk for subsequent sleep problems. Sleep problems during adolescence were not independently associated with subsequent television viewing when prior television viewing was controlled.

In conclusion, sleep plays an important role in the emotional and physical well-being within the pediatric population. As discussed above, fragmented or disrupted sleep causes a variety of clinical issues. An increased understanding more about the symptoms, causes, and implications of interrupted sleep can help us be informed about our situation and find the best treatments or preventative measures to minimize our sleep disturbances.

## Future directions

The relationship between socioeconomic status, environmental factors, and sleep quality is crucial for future research. Exploring how elements such as housing conditions, neighborhood safety, and healthcare access affect children’s sleep and blood pressure can identify key intervention areas. Furthermore, understanding how sleep disturbances with and without OSA lead to pediatric hypertension is crucial for future research. Data from our laboratory have suggested that multiple factors and co-morbidities are associated with hypertension in pediatric patients without OSA.^107^ Utilizing advanced imaging, biomarkers, and longitudinal studies can clarify the involved pathophysiological pathways. Investigating the roles of inflammation, endothelial dysfunction, and changes in the gut microbiome will help determine their effects on blood pressure regulation in children and adolescents. These efforts are crucial to addressing this growing public health concern and improving long-term cardiovascular outcomes.

## Supplementary information

Supplementary Information

## Data Availability

The datasets generated during and/or analyzed during the current study are available from the corresponding author on reasonable request.
