# Supplementary material for: Inadequate sleep is a risk factor for pediatric hypertension: the pathophysiologic and socioeconomic correlates
Source: Pediatr Res. 2025 Jun 25;99(2):470–83. doi: 10.1038/s41390-025-04199-3 (PMC12956601; doi:10.1038/s41390-025-04199-3)
Supplement: Supplementary file 1 — Supplementary Information [file 41390_2025_4199_MOESM1_ESM.pdf]

Supplemental Table 1 (S1): List of Abbreviations and Definitions

Abbreviation	Definition
**OSA**	Obstructive Sleep Apnea
**AHI**	Apnea-Hypopnea Index
**BP**	Blood Pressure
**SBP**	Systolic Blood Pressure
**DBP**	Diastolic Blood Pressure
**PSQI**	Pittsburgh Sleep Quality Index
**PSG**	Polysomnography
**LVH**	Left Ventricular Hypertrophy
**CKD**	Chronic Kidney Disease
**CVH**	Cardiovascular Health
**HMOD**	Hypertension-Mediated Organ Damage
**WASO**	Wake After Sleep Onset
**SOL**	Sleep Onset Latency
**MetS**	Metabolic Syndrome
**AIx75HR**	Augmentation Index (adjusted for 75 heart rate)
**BMI**	Body Mass Index
**eBP**	Elevated Blood Pressure
**SpO2**	Oxygen Saturation
**PH**	Primary Hypertension
**RR**	Relative Risk
**LVMI**	Left Ventricular Mass Index
**SDB**	Sleep-Disordered Breathing
**MAP**	Mean Arterial Pressure
**CBT**	Core Body Temperature
**SF**	Sleep Fragmentation
